# Clinical teaching of university-degree nursing students: are the nurses in practice in Uganda ready?

**DOI:** 10.1186/s12912-020-00528-5

**Published:** 2021-01-04

**Authors:** Amos Drasiku, Janet L. Gross, Casey Jones, Champion N. Nyoni

**Affiliations:** 1 Department of Nursing and Midwifery, Muni University, P.O. Box 725, Arua, Uganda; 2 Department of Nursing, Morehead State University, Morehead, Kentucky USA; 3 Beth-El College of Nursing, University of Colorado, Colorado Springs, 80918 USA; 4 School of Nursing, University of the Free State, Bloemfontein, South Africa

## Abstract

**Background:**

Nurses with degree qualifications offer better nursing care compared to nurses prepared at lower levels. University based nursing degrees have been sanctioned as entry into professional nursing and several low-resource states have introduced university based nursing degrees. The clinical teaching of students enrolled in such degrees is challenged, as most nurses in practice do not have university degrees and may not have the necessary skills to facilitate clinical learning as expected at degree level. A university in Uganda established a bachelor’s degree in Nursing program and was expecting to use nurses in practice at a teaching hospital for the clinical teaching of university-degree nursing students. This study reports on the perceptions of the nurses in practice regarding their readiness for the clinical teaching of undergraduate nursing students.

**Methods:**

A qualitative descriptive research study was conducted among 33 conveniently sampled nurses from Arua Regional Referral Hospital (ARRH) who had been supervising Diploma and/or Certificate in Nursing students. Five focus group discussions and three informant interviews were used to generate the data. Data were transcribed verbatim and analysed using an inductive approach through thematic analysis.

**Results:**

The nurses in practice perceived themselves as ready for clinical teaching of undergraduate nursing students. Three themes emerged namely; “*Willingness to teach undergraduate students*” “*Perceived attributes of undergraduate students*”, and “*The clinical practice environment*”.

**Conclusion:**

The nurses in practice need support in the execution of the clinical teaching role of university-degree nursing students. The nature of supports would include, continuing professional development specific to clinical teaching, engaging the educators in the clinical environment, positively engaging power gradients and address insecurities among the nurses and the students. Students in these programmes should be exposed to the clinical environment earlier within the programme, and be exposed to interprofessional and trans-professional education.

## Background

Nurses with university degrees offer better nursing care when compared to nurses trained in non-university nursing programmes [1]. Several studies, conducted predominantly in high-income countries, have demonstrated that patients cared for by nurses with university degrees have better health outcomes compared to those with other qualifications [2, 3]. According to DeBack & Mentkowski [4] nurses with university degrees have higher level of reasoning, think critically and make sound clinical judgements when executing patient care [5].

The quality of university-based nursing degree programmes is mediated by university-wide quality assurance processes and professional regulatory councils [6, 7]. The robust quality assurance mechanisms ensure university-based nursing programmes meet both educational and professional nursing standards, which is not common practice in non-university based nursing programmes [8]. In addition to the generic nursing science courses, university-based nursing degree programmes integrate basic and social sciences which are usually facilitated by experts. These basic and social sciences, such as Human Anatomy, Physiology, Microbiology, Pharmacology, and Psychology, are quintessential to the reasoning processes inherent in nursing care [9]. Nurse educators facilitating in these university-based nursing degree programmes are also expected to have advanced educational and professional qualifications at a level higher than the qualification they are facilitating [6]. The undergraduate nursing students are taught both in the university, and university/nursing council-accredited clinical practice sites.

University-based nursing degree programmes are not without controversies. The nursing graduates from these programmes have been described as laden with content but without clinical practice and often perceived to struggle integrating theory and practice [10, 11]. Other criticisms are that nurses from university degree programmes struggle to “hit the ground running” and require extensive induction processes [12]. The graduates from such programmes have also complained of the limited clinical exposure, limited clinical accompaniment during training and the lack of structured practical components in their degree programmes [13]. These frustrations have compromised the argument supporting degree-education for nurses resulting in a need for more robust evaluation of university-based nursing degree programmes especially in resource-limited settings like Africa.

Nevertheless, the benefits of having nurses with degree qualifications outweigh the general stakeholder concerns, resulting in various countries developing and implementing university-based nursing degree programmes. Numerous countries have sanctioned university-based nursing degree qualifications as the minimum requirements for professional nursing practice whilst the majority of low and middle-income countries have established university-based nursing degree programmes in addition to other nursing education programmes [14]. Such a stance is perceived as improving the status of nursing further elevating nurses to be at par with their interprofessional colleagues, in addition to the general benefits related to patient health outcomes [5].

University-based nursing degree programmes also have to meet the professional requirements as stipulated by nursing councils. These professional requirements include specific clinical competencies to be met by the undergraduate nursing students in authentic clinical environments under the supervision of suitably qualified and experienced clinical facilitators [6, 15]. The State of World Nursing report indicates that the majority of nurses in practice do not have university-based nursing degrees, especially in low-resource settings [16]. This situation compromises the clinical experience of the undergraduate nursing students enrolled in university-based nursing degree programmes as the nurses in practice may not have the necessary skills to facilitate clinical learning as expected at degree level. Degree level nurses should be able to transfer learning in the clinical environment through demonstrating reasoning, appropriate clinical judgement and application of all relevant sciences, not only psychomotor skills [17]. Nurses in practice may supervise and demonstrate psychomotor skills but may not be competent in facilitating the integration of knowledge and the development of reasoning processes as expected at degree level.

Low-resource settings are further challenged by a dire shortage of nurse educators with advanced educational and professional qualifications [18, 19]. The available nurse educators are usually over-worked and rely on nurses in practice for the clinical teaching of undergraduate nursing students enrolled in university-based nursing degree programmes [20, 21]. We argue that the quality of clinical learning for university-based nursing degree programmes, especially in low-resource settings, may be enhanced by understanding the perceptions of nurses in practice regarding their readiness for the clinical teaching of undergraduate nursing students. In this study, we explored the perceptions of nurses in practice regarding their readiness for clinical teaching of nursing students enrolled in a university-based nursing degree. Various terms have been used in the literature to describe the role of the professional staff nurses in the clinical education of nursing students in the clinical environment. For example, terms employed include mentor, preceptor, clinical teacher, and clinical supervisor yet the meaning of the terms may differ from setting to setting [22, 23]. In low resource settings the nurse in practice is expected to supervise and teach the essential clinical components of nursing in conjunction with their specific clinical duties. The role of the nurse in practice in low resource settings is thus described as being a clinical teacher regardless of their academic preparation or abilities to facilitate the integration of knowledge and the development of reasoning processes as expected at degree level.

## Methods

### Study context and design

The Muni University in Uganda established a 4-year Bachelor of Science in Nursing (BScN) direct-entry degree programme in 2015. The first two pre-clinical years of the programme focus on the basic and social sciences, while the latter two clinical years focus on the application of the nursing sciences in the clinical learning environment. Students enrolled in this programme are predominantly placed at the Arua Regional Referral Hospital (ARRH). ARRH is an accredited teaching hospital and the nurses in practice have been predominantly engaged in the clinical teaching of nursing students enrolled in non-university-based nursing programmes such as the Diploma in Nursing and the Certificate in Nursing. These nurses in practice were expected to facilitate the clinical teaching for university-degree nursing students enrolled in the BScN programme for the first time in August 2018. The nurses would allow the nursing students attached to their wards or units to shadow them, explain and demonstrate skills if there is an opportunity, give the students chance to demonstrate the skill as they observe and then give feedback. Depending on the students’ level of competence the nurses’ role may be supervisory and they then participate in clinical learning assessments. This study used qualitative research in describing the perceptions of nurses in practice regarding their readiness for the clinical teaching of undergraduate nursing students at the ARRH.

### Study participants

The participants of this study were nurses working at ARRH. The nurses’ highest qualifications comprised a Diploma in Nursing and/or Midwifery, a Certificate in Nursing and/or Midwifery and BScN. All the nurses included in this study had been engaged in clinical teaching of nursing students enrolled in other programs for lower qualifications for more than a year. Some of the nurses were serving as unit/ward in-charges. Intern nurses, volunteer nurses and nurses in their probation period of employment were excluded because of short duration of working at ARRH.

### Pilot study

To ensure feasibility of the interview guide and the entire study approach, a pilot or exploratory study was conducted by the first author. Twelve [12] nurses in practice were invited for a focus group discussion, while a nurse with a degree was interviewed as a key informant. These participants matched the participant profile of the final study participants. Secondary to the pilot or exploratory study, the researchers refined the interview guide to enhance clarity and appropriateness.

### Data collection

Secondary to the approval of this study, all the potential participants were invited to participate in this study. In the invitation to the study, the first author, who is an academic staff at Muni University and had worked with the nurses in practice at ARRH, explained the purpose of the study verbally in addition to the information leaflet. None of the invited participants declined to participate or dropped out from the study. Five focus groups (FGs) each comprising six participants and three key informant (KI) interviews were used to collect data between July and September 2018, before the first group of the BScN students was to be placed in the clinical environment. According to Guest et al. [24], 90% of all themes are discoverable within three to six focus groups, therefore five FGs were sufficient for saturation. All the interviews were guided by an interview guide developed by the author (Table 1). All the data were collected in English by the first author who had been trained in conducting interviews in qualitative research. The data were generated from the following central question;

*“What is your perception regarding your readiness for the clinical teaching of the BScN students?”*

**Table 1:** Interview guide, Interview guide for focus group discussions and key informant interviews with practicing nurses.

**Pleasantries**	1. Hello and how are you? 2. Thank you for agreeing to be part of this interview.
**Central Question**	Please describe perception regarding your readiness for the clinical teaching of the BScN students?
**Supporting and Follow up Questions**	1. Please describe your experience in teaching or supervising student nurses in the clinical area during your professional practice. 2. In your opinion, how different do you expect clinical teaching of BScN students to be from that of the lower level student nurses? 3. What barriers or challenges do you perceive that can affect clinical teaching of BScN students at ARRH? 4. What do you suggest should be done to ensure good clinical teaching of BScN students at ARRH?

Additional probing questions arose from the discussions. Data were collected in a private room within the hospital in the presence of a moderator and all interviews were audio recorded. The author also wrote reflective notes during the data collection process. On average, the focus group discussion (FGDs) lasted 40 min while the KI interviews took 45 min.

### Data analysis

The data were analysed through an inductive reasoning approach inspired by a data analysis framework by Saldaña [25]. According to Saldaña [25], qualitative data analysis is executed in three sequential steps. In the initial step, the digitally recorded interviews were transcribed verbatim and the transcripts were assigned specific identifiers. The transcripts were formatted and stored in a password-protected electronic folder. Before coding, the researchers read and re-read the transcripts to understand the general ideas discussed in the focus groups. Initial, open, axial and in-vivo coding methods were used in coding units of analyses in the transcripts. The authors identified the units of analyses as sentences, phrases, and paragraphs that were related to the research question. The first author coded the data and the other authors acted as co-coders. The outcome of this second step was a final code list after a discussion involving all the authors. In the final step of the data analysis, the codes generated in the second step were further analysed through pattern coding methods. The researchers’ clustered codes with similar meaning and the outcome of this step were themes and these are presented as the findings of this study. The units of analysis are presented as verbatim quotes to support the generated themes.

### Rigor

The trustworthiness framework was applied throughout this study [26]. The researchers engaged already established approaches and frameworks in conducting this study. The data were collected by an experienced researcher, while the data analysis was influenced by contemporary frameworks in data analysis, and experienced researchers were used as co-coders. The researcher presented the findings of the study to some of the participants and they provided feedback on the outcome.

## Findings

Thirty-three nurses participated in this study and all of them had been engaged in clinical teaching of nursing students enrolled in other programs seeking lower qualifications for more than a year. The majority of the nurses were female (29, 87.9%), aged 31–40 years (15, 45.5%) and worked for 11–20 years (18, 54.5%). The nurses’ highest qualifications comprised a Diploma in Nursing and or Midwifery (*n* = 18), a Certificate in Nursing and/or Midwifery (*n* = 12) and BScN (*n* = 3) (Table 2).

**Table 2:** Sociodemographic characteristics of the nurses (*N* = 33)

Variable	Number (Percentage [%])
**Age Group (years)**	
≤30	2 (6.1)
31–40	15 (45.5)
41–50	13 (39.4)
51–60	3 (9.1)
**Sex**	
Male	4 (12.1)
Female	29 (87.9)
**Highest level of Qualification**	
Certificate	12 (36.4)
Diploma	18 (54.5)
Graduate (BSN)	3 (9.1)
**Work Experience (years)**	
≤10	9 (27.3)
11–20	18 (54.5)
21–30	5 (15.2)
31–34	1 (3.0)

Data source: 33 nurses who participated in five focus group discussions and three key informant interviews conducted between July and September 2018 at ARRH

Three themes emerged from the data namely, “*Willingness to teach undergraduate students*” “*Perceived attributes of undergraduate students*”, and “*The clinical practice environment*”.

### Theme 1: willingness to teach undergraduate nursing students

The participants in this study expressed the willingness to teach undergraduate nursing students. Their willingness was tied to their perceived professional competence but was hinged on specific influential conditions and the need for formal training on clinical teaching.

The participants felt competent in the clinical teaching of undergraduate nursing students because of their extensive experience as nurses. The participants had also been engaged in the clinical teaching of nursing students enrolled in Diploma and Certificate programmes from other non-university nursing schools within the country. Also, the participants perceived themselves as having extensive practical experience and were aware of their environment which was deemed essential for clinical teaching. Some of the participants stated;

*Personally, I have been in this field for quite a period. My colleagues all seated here practically have knowledge and skills. We are the colonial nurses, we are hands-on. We were trained under the Ministry of Health. (FG 1, Participant 6).*

*What makes me ready is this hospital being a regional hospital we receive so many referrals with different conditions which I feel I will be able to teach them. (FG 3, Participant 3).*

*I feel I am ready because I am working in a regional referral hospital where most of the facilities are available for their teaching. (FG 5, Participant 5).*

*It is not the first time for me to interface with students. This hospital has been training the nurses at certificate and diploma level, and I have found myself in guiding and teaching them. With the added knowledge at bachelor’s level, I can equally be able to guide them well. (Informant 2).* Several conditions were perceived as influential to the willingness of the participants to teach undergraduate nursing students. These conditions focused on undergraduate students and nurse educators from the university. The participants expressed that they were willing to teach the undergraduate nursing students in the clinical environment. These students were expected to be self-directed, able to ask questions, and seek their own learning opportunities.

*I am ready to give them the knowledge, teach them if they are also cooperative and ready to learn. (FG 4, Participant 4).*

*These students need to be able to ask us questions when they don’t understand things in the clinic area … we won’t know if they don’t ask (FG3, Participant 1).*

The participants felt that the clinical teaching of undergraduate nursing students, should not be the sole responsibility of the nurses in practice, but include the nurse educators from the university. A key informant expressed;

“*… let this supervision not be left entirely to the staff of the hospital. The university staff who are teaching them will equally be assigned to follow these students to back us. (Informant 2)*

The nurse educators from the university were further challenged to engage with current clinical nursing practice. The participants felt that the execution of their clinical teaching role would be compromised if nurse educators from the university were not engaged with current clinical nursing practice. Nursing students would be confused by the differences between the classroom-based theory and the expectations in clinical practice. A participant stated;

*Some of the instructors just teach they do not do the practical work. So they keep on teaching the same things every year, you come to the ward, things have changed. You keep on telling the students new things and they tell you our teacher told us this. The teachers in the training schools do not have the current information but for us we do. (FG 1, Participant 2).*

The participants expressed that they had not been trained formally in the mechanisms surrounding clinical teaching of undergraduate nursing students. They stated that they used their own experience when they were students, and what they observe when other health professionals are teaching students. The participants expressed that formal training in clinical teaching would enhance their willingness and effectiveness in teaching undergraduate nursing students. A key informant expressed;

*The training (provided to students in the clinical setting) is just based on the experience of what we have been doing directly but not after being trained specifically on how to train the students in clinical area. We have not got that formal kind of training. (Informant 3).*

### Theme 2: perceived attributes of undergraduate students

The participants in this study expressed their perceptions regarding some of the attributes of the undergraduate nursing students and how such perceived attributes influence clinical teaching within the referral hospital. The undergraduate nursing students were perceived as threatening, lacking interest in clinical practice, and would engage in general hospital misconduct. The participants based their perceptions on their experience with nursing students enrolled in Diploma and Certificate programs who were being placed at this clinical training platform from other national nursing schools.

The participants expressed a fear of being undermined by the undergraduate nursing students. The fact that the students were enrolled in a university-based nursing degree programme threatened the nurses in practice, who predominantly had lower qualifications than what the students were seeking in the present program. These students were perceived as learning advanced content at the university and that the nurses in practice would struggle to facilitate learning in clinical practice resulting in being undermined by the undergraduate students. Some participants explained;

*“… one can make a mistake, maybe not knowing something in-front of them as they are more learned. They may correct you and then feel big, that they know more and then start undermining staff. (FG 3, Participant 6).*

*Whatever little we have known, we would be in a position to give them. When they want to explore more, I expect them to ask questions. Since these undergraduates are more knowledgeable than us, I think when they will be with us on the ward, if they have seen us not telling the right things to them, they should also tell us. (FG 2, Participant 2).*

According to the participants, students enrolled in a university-based nursing degree programme generally lacked interest in clinical practice and learning within the clinical platform. This perceived lack of interest in clinical practice was expected to influence the ability of the nurses in practice to teach these students. The heavy workload for the nurses in practice, where there is limited time for the nurses in practice to follow-up with each student compounded this situation. A participant expressed;

*Some of these students do not show interest. They tend to move away from certain units, moving up and down, not concentrating. So your interest in training them with much workload we tend not also to concentrate on their training. (FG 2, Participant 3).*

The participants further expressed some examples and outcomes related to the perceived lack of interest in clinical practice by undergraduate nursing students. The participants stated that some of the undergraduate nursing students would prefer and choose specific clinical duties over others, avoid certain clinical platforms and eventually be part of general hospital misdemeanours.

*Sometimes the barrier is dodging of duties like in theatre. It is sometimes busy and some people don’t like the work there like the sluice room. Others start to dodge their duties. (FG 4, Participant 6)*

### Theme 3: the clinical practice environment

The participants in this study described the clinical practice environment as an enabler and barrier to authentic clinical teaching of undergraduate nursing students. On one hand, the participants described aspects of the clinical learning environment that enabled their role in facilitating learning among undergraduate nursing students. The referral hospital receives advanced cases from other hospitals or health facilities within the geographic region, presenting an opportunity for the students to experience advanced nursing care.

*This is a Regional Referral Hospital (RRH), I think all the conditions are there, the patients are overwhelming in all the units, and there are even rare conditions which they can learn about. (Informant 3)*

The availability of specialist services within the hospital setting including specialist doctors and other health service providers provided an opportunity for students to learn from other health professionals. The nurses in this study expressed that the other health professionals always seemed eager to teach students using several opportunities like grand ward rounds.

*We have specialists to guide them in different professions. We can line up with them and transfer better knowledge for them. (FG 2, Participant 3)*

On the other hand, the participants in this study reflected on barriers within the clinical practice environment that would influence their ability in teaching undergraduate nursing students. An example was the shortage of hospital equipment, which was perceived to influence the participants’ ability to teach undergraduate nursing students ideal nursing care and clinical practice.

*Some of the hospital equipment which you need to show practically are not there. You will also teach them the theory and yet they have been sent to see some of the hospital equipment practically. (FG 4, Participant 4).*

In addition to the shortage of equipment, the participants in this study described the shortage of nurses in practice. The nurses in practice are faced with a heavy patient load and prioritise patient care over the clinical teaching of undergraduate nursing students. The participants expressed;

*In the hospital here we are very few; we have that challenge of not really mentoring these students properly. Sometimes you are alone on the ward and it will be very difficult for you to really monitor these students very carefully because our number is really few. (FG 3, Participant 5).*

*When you are so busy, and you have a long queue [of patients], you have no time to teach these students because you are looking at completing the queue [of patients], at the end of the day, so that will block you from teaching the students. (FG 2, Participant 4).*

## Discussion

This study sought to describe the perceptions of nurses in practice regarding their readiness for the clinical teaching of nursing students enrolled in a university-based nursing degree programme in Uganda. The participants in this study, who were nurses in practice at one teaching hospital, generally perceived themselves to be ready and willing to be engaged in the clinical teaching of undergraduate nursing students. Their readiness for clinical teaching was linked with their perceived competence, the perceived attributes of undergraduate students and the clinical practice environment.

Most of the nurses in practice in low-resource settings such as Uganda have professional qualifications below the university-degree [16]. However, these nurses have invaluable professional experience and expertise which are fundamental to the clinical teaching of undergraduate nursing students [10]. In this study, the participants perceived themselves to have adequate nursing experience for the clinical teaching of undergraduate nursing students. However, these nurses claimed not to have been formally trained in clinical teaching. Mkony et al. [27] explained that the lack of training in clinical teaching erodes the value of their clinical experience and expertise, negatively influencing the quality of clinical education of undergraduate nursing students. The literature further describes several disadvantages of content-based education, which include content that is not based on practical realities, overloaded curricula and nurse educators who are distant from the clinical practice environment [28]. In this study, the nurses in practice expressed the need for nurse educators to be more involved with the clinical teaching of their students and to be up-to-date with current nursing practice. In their argument for competency-based nursing education (CBE), Gruppen, Mangrulkar, and Kolars [29] explain that CBE is driven by professional competencies that enable the integration of clinical practice and clinically driven assessments within a nursing programme. Integrating CBE in the BScN programme at the Muni University will inevitably force nurse educators to be updated with the current clinical practice, engage in best available evidence and drive for clinically driven assessments [30]. In addition, this would improve the presence of nurse educators within the clinical practice environment hence improving the quality of clinical education for undergraduate nursing students. However, with this approach, educators may feel challenged and incompetent due to lack of updated clinical skills.

Students enrolled in a higher qualification program may be perceived as threatening and intimidating to teach. The participants in this study revealed their fear of being undermined by undergraduate students. Research confirms fear and intimidation as a barrier to authentic learning for nursing students and genuine clinical teaching by the nurses in practice [31–33]. Rebeiro, Edward, Chapman, and Evans [34] explains the origins of such fear as linked to power dynamics amongst the nurses in practice, who ordinarily leverage on their experience and expertise as power over nursing students. The fact that the undergraduate nursing students are enrolled for a qualification higher than the nurses in practice threatens this power dynamic and is evidenced by the general fear and intimidation [35]. The nurses in practice may retaliate to this perceived threat to their power through creating a hostile environment for learning, ignoring undergraduate nursing students and in some cases executing unnecessary punitive measures. Halman, Baker, and Ng [36], influenced by the critical social theory, suggests the need for detangling power dynamics in health professions education through critical consciousness. Role players in the clinical practice environments, namely the undergraduate students and the nurses in practice, are expected to consciously engage in their inherent power and how such power influences the other role players and the clinical practice environment. Such conscious engagement should lead to value clarification aimed at destabilising hierarchies and hegemony in the clinical practice environment and creating grounds of symbiotic relationships and mutual respect. The nurse educators should facilitate such discussions during clinical practice orientation programmes for the undergraduate students and the nurses in practice and remain vigilant and sensitive to such power gradients throughout clinical placements.

Advances in medicine and general approaches to healthcare such as the adoption of the primary healthcare approach have influenced the numbers and types of patients in hospitals, their lengths of hospital stays and the mix of healthcare workers within hospitals. These factors have influenced programmes planners for nursing programmes to re-evaluate the value and benefits of clinical placement sites in relation to the expected clinical competences. In this study, the clinical placement site was perceived to have a high number of patients with various health conditions and also a larger pool of health professionals who can be involved in the clinical teaching of undergraduate students. This setting is ideal for undergraduate student learning and has opportunities for interprofessional and trans-professional education. Bjørke and Haavie [37] warn that the other non-nursing professionals need to be aware of the expected professional and educational outcomes of the undergraduate nursing students before they can be engaged to teach them. Awareness of the professional and educational outcomes enables the non-nursing facilitators to pitch their educational sessions appropriately for the benefit of the undergraduate nursing students.

Typical of resource-limited environments is the lack of resources to facilitate clinical teaching. This lack of resources has been demonstrated to affect the quality of clinical teaching and the overall clinical education of all health professions education programmes. The participants in this study perceived a lack of resources for clinical teaching which influenced their ability to teach undergraduate nursing students. Linegar, Whittaker, and van Zyl [38] reflect on the essentiality of training institutions or teaching hospitals to be accredited using a set of minimum accreditation standards that reflect clinical resources. Only accredited institutions would then be used for the clinical teaching of students. However, Bates, Schrewe, Ellaway, Teunissen, and Watling [39] explain that some resources would never be available and further implore nursing education institutions to engage contextual driven-programmes. In his argument for contextually driven-programmes, Obadeji, A [40]. states that in addition to designing health professions programmes aligned with the local disease profile, education institutions should also design programmes based on locally available resources. Contextually driven-programmes move away from Western-generated or global prototypical programmes which often create gold standards that are not based on contextual realities.

## Conclusion

University-based nursing degree programmes have been demonstrated to produce graduates who can positively influence the health outcomes of the patients under their care. In as much as these programmes are underpinned by strong quality assurance mechanisms within the university and from professional regulation, the clinical teaching within such programmes remains a challenge within low resource settings [41]. In these settings, the nurses in practice do not have university degrees but are expected to be engaged in the clinical teaching of the nursing students enrolled in university-based nursing degrees.

Through qualitative research, we explored the perceived readiness of nurses in practice for the clinical teaching of students enrolled in a University-based nursing degree programme. In as much as the nurses in practice perceived themselves to be ready to engage with the students enrolled at university-based degree programmes, several other issues were expressed as influencing their perceived readiness. These issues included their concerns about their personal nursing competence, the perceived attributes of the students and the clinical practice environment.

The findings of this study may have been influenced by several limitations, namely that only nurses in one placement site were used to inform the findings of this study. The voices of other health professionals involved in the clinical teaching of nursing students could have been explored to enhance the quality of the study findings. Nevertheless, low- and middle-income countries must promote quality education of nurses and the introduction of university-based nursing degrees offers opportunities to improve health outcomes and improve the status of nursing. The nurses in practice bear the potential of influencing and improving the quality of the education of students enrolled in university-based nursing programmes.

Several recommendations are made to proponents of university-based nursing degree programmes in low resource settings, namely that;

- Continuing Professional Development programmes tailor-made to the nurses in practice regarding clinical teaching must be developed and implemented;
- Competency-based nursing education grounded on contextual realities and needs should replace content-driven programmes and underpin the design and development of undergraduate nursing programmes to enhance the practical abilities of educators and students;
- Students in university-based degree programmes should be exposed to the clinical environment earlier within their programmes, and
- Universities must harness and fortify opportunities for interprofessional and trans-professional education for their undergraduate students.

## Data Availability

All data generated or analysed during the current study are available from the corresponding author on reasonable request.

## Abbreviations

ARRH: Arua Regional Referral Hospital
BScN: Bachelor of Science in Nursing
CPD: Continuing Professional Development
CBE: Competency-based Nursing Education
UNFPA: United Nations Population Fund
FGs: Focus groups
FGDs: Focus group discussions
KI: Key Informant
